# The Effect of Ballistic Exercise as Pre-Activation for 100 m Sprints

**DOI:** 10.3390/ijerph16101850

**Published:** 2019-05-24

**Authors:** Maria H. Gil, Henrique P. Neiva, Nuno D. Garrido, Felipe J. Aidar, Maria S. Cirilo-Sousa, Mário C. Marques, Daniel A. Marinho

**Affiliations:** 1 Department of Sport Sciences, University of Beira Interior, 6201-001 Covilhã, Portugal; maria.helena.gil@hotmail.com (M.H.G.); henriquepn@gmail.com (H.P.N.); mariomarques@mariomarques.com (M.C.M.); 2 Research Center in Sports Sciences, Health Sciences and Human Development, CIDESD, 6200-001 Covilhã, Portugal; ndgarrido@gmail.com; 3 Department of Sports, Exercise and Health Sciences, University of Trás-os-Montes e Alto Douro, 5001-801 Vila Real, Portugal; 4 Department of Physical Education, Federal University of Sergipe - UFS, São Cristovão, SE 49100-000, Brazil; fjaidar@gmail.com; 5 Post Graduate Program in Master’s level in Physical Education, Federal University of Sergipe-UFS, São Cristovão, SE 49100-000, Brazil; 6 Post Graduate Program in Doctorade and Master’s level in Physiological Sciences, Federal University of Sergipe - UFS, São Cristovão, SE 49100-000, Brazil; 7 Group of Studies and Research of Performance, Sport, Health and Paralympic Sports - GEPEPS, the Federal University of Sergipe - UFS, São Cristovão, SE 49100-000, Brazil; 8 Associate Graduate Program in Physical, Department of Physical Education, Federal University of Paraíba, João Pessoa, PB 58051-900, Brazil; helpcirilo@yahoo.com.br; 9 Department of Physical Education, Regional University of Cariri, Crato, CE 63105-010, Brazil

**Keywords:** warm-up, performance, repeated-sprint, physiology, biomechanics

## Abstract

The benefits of warm-up in sports performance has received a special interest in the current literature. However, there is a large gap of knowledge about the tasks to be performed, specifically in the real competitive environment. The purpose of the study was to verify the acute effects of a warm-up including ballistic exercises in 100 m running performance. In addition, a second 100 m trial was assessed to better understand the warm-up effects in training and competition. Eleven men (25.4 ± 6.2 years of age, 1.76 ± 0.08 m of height, 78.2 ± 8.6 kg of body mass) were submitted to three different protocols, in a randomized order: no warm-up (NWU), typical warm-up (WU) and WU complemented with ballistic exercises (PAP). Biomechanical, physiological and psychophysiological variables were assessed. Differences were found between the three conditions assessed in the first 100 m sprint with 7.4% and 7.6% faster performances after the WU and PAP, compared to NWU. Stride length was higher in the second part of the 100 m after PAP compared with WU. These results highlight the positive effects of warm-up for sprinting performance. The inclusion of ballistic exercises, besides being used to improve sprint performance, can increase stride length in the final of the 100 m race.

## 1. Introduction

Warm-up practices have been used to prepare the athlete for training and/or competition [1]. It is believed that a well-designed warm-up causes physiological changes and helps the athlete to increase the mental focus for the next task, allowing them to optimize the performance [2]. The main effects of warming up derived from increased body temperature and from the muscle movement, both contributing to decreased joint and muscle stiffness, improved nerve conduction rate, efficient metabolic reactions, increased blood flow to the active muscles, increased oxygen uptake and to potentiate post-activation mechanisms [3,4].

There has been an increase in interest in the warm-up issue, evidenced by the number of recent studies and most reporting high benefits for performance in different sports and activities [5,6,7]. Specifically, in running, it is now well stablished that warm-up improves sprint performance [1,6,8]. Usually, warm-up included a brief period of low intensity aerobic (e.g., light to submaximal running) and stretching exercises, followed by specific exercises related with the following activity and/or sport [1,9]. During this specific phase of warm-up, the coaches and the athletes have been using several exercises for the same purpose, such as dynamic and static stretching [10], agility exercises and ballistics [11]. The last of these, the ballistic exercises, are a recent trend of specific warm-up and are believed to cause a post-activation potentiation phenomenon, thus enhancing the performance [12,13].

Researchers have looked at the post-activation potentiation phenomenon, suggesting that it might improve muscle power manifestations [14,15]. This increase in force production usually happens after a maximum or near maximal muscle stimulation [10]. Post-activation potentiation seems to augment muscle force generating capacity as a result of the previous contractile history of the muscle cells involved in the previous contraction [14]. There is an acute effect that increases the speed of conduction of the nerve impulse to the muscle, increases the number of recruited motor units and improves the interaction mechanism of contractile filaments [16]. The main mechanisms responsible for this are not totally clear, but studies attributed improvements to the increased phosphorylation of the myosin regulatory light chain [17,18,19]. Post-activation potentiation seems to cause neuromuscular changes and improves type II muscle fiber activity, thus favoring performance in short-term maximal efforts [13].

An improvement of 3% was found in 40 m sprints after performing back squats at 85% of 1 repetition maximum (1RM) [20]. Improvements of 2% and 3% were also found in 10 and 30 m sprints after 10 repetitions of half back squat exercise at 90% 1RM [21]. Nevertheless, Kilduff et al. [22] found that one set of three repetitions of a squat exercise at 87% 1RM did not improve 15 m swimming performance, compared to a traditional in-water warm-up. Previous research mainly focused on high external loads of strength exercise during warm-up and it is known that it cannot be applied in a real competition context [13,20,23]. There is a real need for understanding the effects of the post-activation potentiation using some usual tasks that can be reproduced in a real competition venue. The first studies on this revealed that including depth jumping in the warm-up protocol increased both maximal strength [24] and vertical jump [25,26]. Byrne, Kenny and O’Rourke [27] concluded that the addition of three depth jumps resulted in a 5% improvement of 20 m running compared to a traditional warm-up. However, little is known regarding when these ballistic exercises are used before Olympic racing distances, such as the 100 m. Moreover, little is known about the effects of using post-activation potentiation strategies on the biomechanical variables during running. Running performance depends on the stride parameters and, for instance, the optimal ratio between stride length (SL) and stride frequency (SF) enable maximal sprinting velocity and efficiency [28]. This relationship is conditioned by the neuromuscular regulation of movement, morphological characteristics, motor abilities and energy substrates [29,30], all of which can be influenced by warm-up tasks [1,7,13].

Therefore, it was hypothesized that a warm-up that included ballistic exercises would improve 100 m running performance, by changing the stride parameters (SL and SF) and physiological response. So, the primary aim of the current study was to verify the acute effects of a warm-up including ballistic exercises inducing a post-activation potentiation, easy to apply in a real competition context, in 100 m running performance. In addition, a second 100 m trial was assessed to better understand the warm-up effects during competition and training. To the best of our knowledge, no previous investigation has used a second repetition, and this is important to understand the neuromuscular and metabolic responses, helping to develop optimized training strategies. Repeated efforts have been used as determinants for success in a wide range of sports and may be associated with neuromuscular and metabolic factors that influence performance [31,32]. The primary outcomes for our study were the 100 m running performance (time) and biomechanical variables (SL and SF). Secondary outcomes included physiological (lactate concentration and heart rate) and psychophysiological (ratings of perceived effort) variables.

## 2. Materials and Methods

### 2.1. Participants

Eleven men aged 20–36 years (mean ± SD: 25.4 ± 6.2 years of age, 1.76 ± 0.08 m of height, 78.21 ± 8.59 kg of body mass) volunteered to participate in this study. Participants were physically active sport science students. Each individual was asked to report any previous illness, injury or other physical issue that would hinder their performance. Participants were included on the basis that they were healthy, injury free, and engaged in physical activity regularly with an experience of running and testing for the last two years, although they were not competitive sprinters. Criteria of exclusion from the study was the evidence of any medical or orthopedic problem, a self-reported fitness classification below moderately active, or any other self-reported issue that would endanger their own health (assessed via questionnaire). After local ethics board approval, ensuring compliance with the Declaration of Helsinki, the subjects were informed about the study procedures, and a written informed consent was signed.

### 2.2. Design

The purpose of the present study was to evaluate the effects of typical warm-up procedures (WU), the inclusion of post-activation potentiation exercises (PAP) and no warm-up (NWU) on 100 m running performance, analyzing biomechanical, physiological and psychophysiological variables.

Each participant completed two 100 m time-trials after each warm-up condition, in a randomized order, separated by 48 h. The WU design was based on literature recommendations [8,32] and included a low intensity aerobic component followed by specific running tasks. The PAP protocol included lower body ballistic exercises according to previous suggestions [33] after completing WU. During the NWU condition, the subjects were asked not to perform any type of action or movement prior to the 100 m sprint, remaining seated for 5 min. This design was able to test whether the inclusion of PAP strategies during warm-ups affected running performance.

### 2.3. Experimental Procedures

All the procedures took place at the same time of the day (8:00–12:00 a.m.) for each participant under the same environmental conditions (~22 °C air temperature and ~60% of humidity) in an athletics track facility. The participants were familiarized with the warm-up procedures 72 h before the experiments, and they were reminded to maintain the same routines during the assessment days, avoiding strenuous exercise, and abstaining from consuming caffeine 48 h before testing.

**Figure 1:**
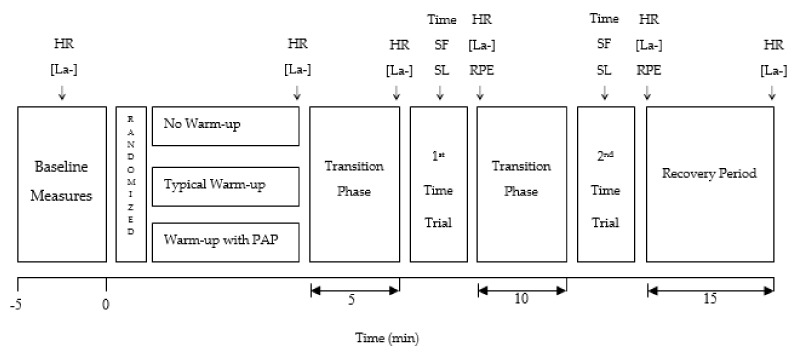
Schematic representation of the study design and testing procedures used. HR = heart rate; [La^−^] = blood lactate concentration; SF = stride frequency; SL = stride length; RPE = ratings of perceived exertion.

After arriving, each participant remained seated for 5 min and baseline measurements of heart rate (HR; Vantage NV; Polar, Kempele, Finland) and blood lactate concentrations([La^−^]; Lactate Pro LT 1710; Arkray Inc., Kyoto, Japan) were then assessed. Each volunteer was then randomly assigned to a warm-up protocol (Figure 1).

### 2.4. Warm-Up Protocols

The warm-ups were designed based on research [1,8,32] and with the help of an experienced coach. The main difference between the warm-ups were the inclusion of lower-body ballistic exercises to stimulate post-activation potentiation. WU comprised 5 min of easy run (lower than 65% of estimated maximal HR), eight exercise drills (20 m repetitions with 10 s of recovery between them), such as rhythmic jumps from foot to foot, ankle drills, skipping drills, high-knee running. Then, these technical exercises were followed by 2 × 40 m running at gradually increasing intensity. In the PAP condition, the participants performed the WU followed by 2 sets of 5 depth jumps from a box of 70 cm height (3 min recovery) as suggested by Maloney et al. [33]. Each jump was performed by stepping off a box with one foot, landing with bent knees, then immediately jumping with maximal effort. The subjects were instructed to jump as quick and high as possible and to keep their hands on their hips to eliminate any contribution of arm swing [9,27].

### 2.5. Time-Trial Performance

Once the participants finished warming-up, they remained seated for 5 min before performing the 100 m time-trials. The subjects started from a standing position with the trunk bent forward and the lower limbs apart and slightly bent, positioned behind the starting line. After official commands, each participant started maximal running using a standing start with the lead-off foot placed 1 m behind the first timing gate. Times were measured by photocell timing gates (Brower photocells, Wireless Sprint System, USA) placed at 0, 50, and 100 m so that the times needed to cover 0–50 m (T0–50), 50–100 m (T50–100) and 0–100 m (T100) could be determined. After 10 min rest, the subjects performed a second 100 m sprint. 

### 2.6. Kinematics

All the procedures were recorded by two video cameras (Casio Exilim Ex-F1, f = 30 Hz) placed perpendicular to the running track. This enabled the acquisition of basic kinematic data such as the number of strides performed by each subject, the average stride length (SL) and average stride frequency (SF) calculations, between 0 and 50 m and between 50 and 100 m, using an open-source software (Kinovea, version 0.8.15). In running, a stride is defined as the time between two consecutive specific discrete events, normally defined as two consecutive foot strikes on the same foot. SL is defined as the distance traveled during a stride and SF is defined as the rate of strides per min. SF was converted to International System Units (Hz) for further analysis. Knowing the time performed and thus the running velocity, SL was determined from the division of running velocity by SF [34,35].

### 2.7. Physiological and Psychophysiological Variables

Capillary blood samples for [La^−^] assessment were collected from the fingertips before and 5 min after warm-ups, 3 and 6 min after each 100 m sprint to obtain the highest value ([La^−^]peak) [36], and after 15 min of recovery. HR was assessed before and after each warm-up (5 min), immediately after each time-trial (1 min) and after 15 min of recovery. Additionally, the rating of perceived exertion (RPE) was recorded using a 10-point modified Borg scale (Borg [37], modified by Foster et al. [38]) after warm-ups and after the time-trials. 

### 2.8. Statistical Analysis

Standard statistical methods were used for the calculation of mean ± SD, and 95% confidence intervals for all variables. The normality of all distributions was verified using Shapiro–Wilk tests. Data for all variables analyzed were homogeneous and normally distributed. The effect of the warm-up procedures was analyzed by an ANOVA for repeated measures, with sphericity checked using Mauchly’s test. When the assumption of sphericity was not met, the significance of *F*-ratios was adjusted according to the Greenhouse–Geisser procedure. Bonferroni post-hoc analysis were performed to further investigate the effect of each condition. All these statistical procedures were performed using IBM SPSS Statistics for Windows^®^, version 22.0 (Armonk, NY, USA: IBM Corp.) and the level of statistical significance was set at *p* ≤ 0.05. In addition, the effect size was calculated to estimate variance between conditions (partial eta squared: η_p_^2^) and Hedges’ g (ES) for within-subjects’ comparisons using the Excel spreadsheet by Lakens [39]. ES values of 0.20, 0.60, 1.20 and 2.00 were considered small, moderate, large and very large magnitudes, respectively [40]. For η_p_^2^, cut-off values were interpreted as 0.01 for small, 0.09 for moderate and 0.25 for large.

## 3. Results

Before warm-up, the physiological variables were not different between conditions. Baseline measurements of HR (70 ± 7 bpm vs. 69 ± 7 bpm vs. 70 ± 7 bpm; *F* = 0.35, *p* = 0.71, η_p_^2^ = 0.04) and [La^−^] (2.5 ± 0.6 mmol∙L^−1^ vs. 2.5 ± 0.6 mmol∙L^−1^ vs. 2.5 ± 0.6 mmol∙L^−1^; *F* = 0.41, *p* = 0.67, η_p_^2^ = 0.04) were similar between the three conditions. 

Table 1 presents a comparison between the HR and the [La^−^] immediately after the warm-ups. It was possible to verify significant differences in HR (*F* = 19.80, *p* < 0.001, η_p_^2^ = 0.69) and [La^−^] (*F* = 35.29, *p* < 0.00, η_p_^2^ = 0.80), with higher values for either warm-ups compared with no warm-up condition. No differences were found in perceived exertion between warm-ups performed (WU: 4.27 ± 1.27 vs. PAP: 3.80 ± 1.40; *p* = 0.34, ES = 0.32).

Table 2 presents the results recorded in the first 100 m sprint after NWU, WU and PAP. Large differences were found between the three conditions assessed (*F* = 12.52, *p* = 0.005, η_p_^2^ = 0.58) in the 100 m sprint. The participants were 7.44% and 7.57% faster after the WU and PAP, compared to NWU, respectively. Moreover, four of them were faster after WU and seven were faster after PAP.

Warm-ups assessed resulted also in large effects in the SF during the first 50 m (*F* = 3.81, *p* = 0.07, η_p_^2^ = 0.30) and the second 50 m (*F* = 9.29, *p* = 0.01, η_p_^2^ = 0.51) of the time-trial. The SL showed to be clearly different only in the second 50 m of the time-trial (*F* = 4.14, *p* = 0.03, η_p_^2^ = 0.32). After trial, no significant differences were found in [La^−^] values (*F* = 2.16, *p* = 0.14, η_p_^2^ = 0.19), HR (*F* = 1.20, *p* = 0.32, η_p_^2^ = 0.12) and RPE values (*F* = 0.18, *p* = 0.73, η_p_^2^ = 0.02).

**Figure 2:**
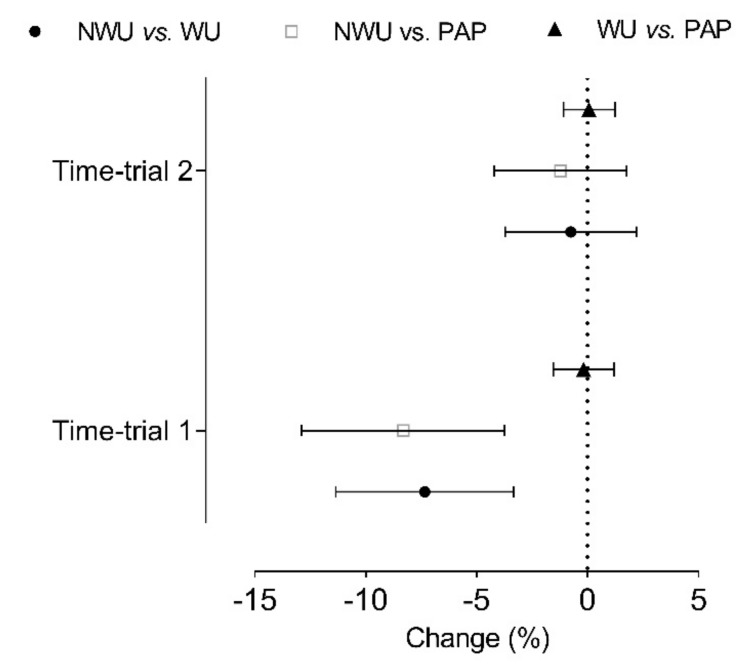
Mean changes (±90% CI) verified between conditions, specifically without warm-up (NWU), after typical warm-up (WU) and after WU complemented with ballistic exercises (PAP) in each 100 m time-trial.

In the second 100 m sprint (Table 3), no differences were found between warm-ups condition (*F* = 0.58, *p* = 0.50, η_p_^2^ = 0.06). Nevertheless, we verified that there was a 6.12% improvement from the first to the second sprint of 100 m in the NWU condition, while the same did not occur in the other conditions. The different responses to each warm-up condition in the 100 m time trials can be easily confirmed in Figure 2.

No significant differences were found in running kinematics during the second sprint, specifically regarding the SF in the first (*F* = 1.89, *p* = 0.18, η_p_^2^ = 0.17) and second 50 m (*F* = 0.33, *p* = 0.72, η_p_^2^ = 0.04), and regarding the SL in the first (*F* = 2.19, *p* = 0.14, η_p_^2^ = 0.19) and second 50 m (*F* = 0.68, *p* = 0.52, η_p_^2^ = 0.07). No significant differences were found in [La^−^] values (*F* = 2.83, *p* = 0.09, η_p_^2^ = 0.24), HR (*F* = 1.21, *p* = 0.32, η_p_^2^ = 0.12) and RPE values (*F* = 0.18, *p* = 0.73, η_p_^2^ = 0.02) after the second time trial.

No differences were found after 15 min of recovery in the HR (NWU: 94 ± 8 bpm vs. WU: 97 ± 18 bpm vs. PAP: 96 ± 6 bpm; *F* = 0.17, *p* = 0.84, η_p_^2^ = 0.02) and in the [La^−^] values (7.8 ± 1.3 mmol·L^−1^ vs. 7.9 ± 1.3 mmol·L^−1^ vs. 7.7 ± 1.0 mmol·L^−1^; *F* = 0.37, *p* = 0.70, η_p_^2^ = 0.04). 

## 4. Discussion

The main purpose of the current study was to verify the acute effects of a warm-up including ballistic exercises, easy to apply on a real competition context, in 100 m running performance. It was intended to benefit from some post-activation potentiation, and thus optimize sprint running performance. This hypothesis was partially confirmed by the increased performance verified in the first sprint compared with the non-existence of warm-up. Nevertheless, by including some post-activation potentiation strategies such as the ballistic exercises, there were no additional effects in performance compared to the typical warm-up procedures. These results are in accordance with previous scientific evidence that reported optimized sprint performances after a typical warm-up or a post-activation potentiation warm-up (e.g., [23,41]) but failed to evidence additional improvement in performances after the use of ballistic exercises, as expected (e.g., [42,43]). Both warm-ups resulted in higher SF in the second part of the first time-trial compared with no warm-up. Interestingly, SL was higher in the second part of the 100 m after PAP compared with WU. This suggest that there are some specific technical adaptations that occur in response to different warm-up stimulations. 

The warm-up is intended to optimize the athletes’ preparedness, by increasing temperature, blood flow and muscle and metabolic efficiency to produce faster responses which are determinant to performance [1,7]. The ability of the muscle to produce force can be acutely modified by warm-up by including some conditioning muscle contractions [22]. The post-activation potentiation elicits transient improvements in performance and has been investigated as a strategy to include during warm-up for increasing performance [23,41]. The common exercises related to potentiation post activation phenomenon have used heavy-load (75–95% 1RM) resistance exercise [23]. However, ballistic exercises can be used as alternative since these are usually related with type II motor unit recruitment [44]. In fact, ballistic activities are more practical and feasible before competition compared to exercises requiring high-intensity external loads. That was the main reason for the assessment of ballistic exercises during warm-up in the current studies. Recent studies found some benefits by using depth jumping during the warm-up protocol to both maximal strength [24], sprint performance [42] and vertical jump [26,42]. However, to the best of our knowledge, no studies evaluated this warm-up strategy when applied to official running distances such as the 100 m race and tried to understand the biomechanical responses during the race. 

The results showed that the 100 m running performance was positively influenced by the warm-up. All the participants performed better after either warm-ups and, despite no statistically significant differences found between WU and PAP (*p* = 1.00, ES = 0.01), seven athletes recorded their best times after PAP. This could mean that there might be an individual response to PAP stimulation, as already highlighted by Till and Cook [42]. These authors found no differences in 20 m running performance by adding different post-activation potentiation strategies to usual warm-up, such as deadlift (5 repetitions at 5 repetitions maximum), or tuck jump (5 repetitions), or isometric maximum voluntary knee extensions (3 repetitions for 3 s) [42]. Nevertheless, others found positive effects on the use of ballistic exercises in running performance. Byrne et al. [27] verified that a brief warm-up of 5 min of running, dynamic stretches and three vertical jumps resulted in 5% better performance in 20 m sprint compared to the warm-up without the jumps. Accordingly, Lima et al. [45] found that 2 × 5 jumps from a height of 0.75 m caused 2% faster 50 m sprint performance. More recently, Turner et al. [44] found that the utilization of alternate-leg plyometric bounding provided an effective strategy for acutely improving sprint acceleration performance (10 and 20 m). Thus, it would be expected that there would be greater differences between the warm-ups performed, since the use of ballistic exercises during warm-up have been suggested as potentiating performance in explosive and short-term efforts [14,15]. In fact, most studies looked at race distances markedly lower than that used in the current study. This longer distance might have caused the potentiation effects to disappear among other determinants of performance [46].

Maximal running performance results from an optimal ratio between SF and SL [28]. Some studies claimed SL to be the most influencing variable for maximal running velocity [47] while others suggested the SF [28]. Nevertheless, it is a fact that the runners adjust the SL and the SF to run most efficiently, optimizing velocity according to their own characteristics [48]. In the current study, better sprint times after warm-up could be caused by the ability to maintain a higher SF on final 50 m of the 100 m sprint without compromising the SL values. This situation did not occur in the NWU condition. Our results corroborated with previous research that suggested that there is a biomechanical adaptation in response to different warm-up procedures [2,49]. 

Interestingly, the running kinematics showed to be different in response to WU or PAP. In the PAP condition, the participants showed greater SL in the beginning of the race, contrarily to the SF that showed to be lower, compared with the WU condition. The PAP seemed to acutely stimulate the force required for an increased SL and perhaps improving the efficiency of the movement, that remained higher in the beginning of the second sprint. The effects of warm-up on acute motor learning and on sensorimotor responses could lead to different biomechanical movement patterns after different warm-ups [49]. Our WU ended with some specific running exercises and the PAP ended with jumps. It is a fact that the running exercises and running acceleration exercises could have prepared the participants to perform higher SF, while the jumps generated a greater capacity to exert muscular power, hence more effective force in less time and thereupon greater SL. So, this different biomechanical running adaptation might be partially explained by the specificity of the preload stimulus, since the vertical jump is biomechanically different from horizontal running. 

The physiological variables showed an increased response to warm-up, with higher HR and [La^−^] values after warm-up, and within the range of values that some authors suggested to be adequate for a proper warm-up [49,50]. This perhaps explain the better response in the first sprint after either warm-up procedures. Nevertheless, those differences disappeared after the first time-trial, which may be seen as a specific warm-up stimulus that in some way places the participant at a similar preparation level. The first sprint enhanced the neuromotor excitability that resulted in performance optimization in a second 100 m sprint [6,31]. The non-existence of differences between HR and [La^−^] values might suggest that PAP stimulation by the ballistic exercises used were not enough to induce physiological stress. Once again, this could be caused by the lack of specificity of the jumps and/or an insufficient load to stimulate some higher responses in PAP. We should be aware of a possible individualized effect of PAP stimulation, that was already documented before [15,51]. Moreover, we could speculate that the interval after PAP was not adequate for each runner [52,53]. It is known that the PAP effect may last for 5 to 10 min [53] and within this period, there are different moments of maximal potentiation for each individual [23]. However, our results were reliable and enlightening about the use of both warm-up procedures and that PAP could be used as an alternative to traditional warm-up.

Some limitations, however, should be addressed. In fact, our results could not entirely be extrapolated to performance of higher skilled sprints during official events since the participants were not sprint specialists/athletes and it is known that post-activation potentiation could be influenced by training levels [19]. Also, further studies should include a larger number of participants and include females to clarify some of the analyzed findings. However, we took several steps to strengthen our statistical analysis as described in the statistical section. Future research should investigate different post-activation potentiation strategies (e.g., combining different jumps or short-term sprints) and different recovery times between the warm-up and the race. Moreover, other evaluation methods could be used to complement our measures and to deepen our findings, such as body temperature and other biomechanical variables (e.g., contact time and horizonal forces production). Considering our limitations, readers should interpret our results with discernment. Even so, the current findings are still relevant for coaches and researchers for increased knowledge on warm-up and the effects on performance. 

## 5. Conclusions

The results suggested that 100 m running performance was positively influenced by warm-up procedures, evidenced by the best results after the WU and the PAP compared to the NWU condition. Moreover, our results suggested that 100 m is equally optimized after WU or PAP, but with different running kinematics. Thus, in support of our original hypotheses, we have demonstrated that warming up benefits the 100 m running performance and that ballistic exercises, easy to perform by using body mass, can be used as an alternative to typical warm-up procedures. 

Some practical applications can be drawn. It seems clear that 100 m sprinters should warm-up for better competitive and training performances. When no warm-up is possible, a single 100 m trial can be enough to stimulate and prepare the athlete for that unusual situation. Yet, it is usually possible to warm-up before the race or training session and in this case, the PAP could be included in the warm-up to potentiate some individual benefits. Moreover, if the individual 100 m race strategy depends on having a higher SF, a typical warm-up should be used, whereas if higher SL is needed, the warm-up including ballistic exercises should be used. The current results alert coaches and researchers the need for tailored and customized warm-up designs and specifically post-activation potentiation strategies during warm-up. The current study took a novel approach to warm-up research by examining the effects of including post-activation potentiation exercises (i.e., ballistic exercises) in running performance and in running stride kinematics. 

## Figures and Tables

**Table 1:** Mean ± SD values (95% confidence interval) of physiological responses to no-warm-up (NWU), typical warm-up (WU) and post-activation potentiation warm-up (PAP) (n = 11). *p*-values and effect sizes (ES) are also presented.

				NWU vs. WU		NWU vs. PAP		WU vs. PAP	
	NWU	WU	PAP	*p*-Value	ES	*p*-Value	ES	*p*-Value	ES
HR (bpm)	72 ± 6 (68, 76)	99 ± 13 (89, 108)	91 ± 9 (86, 97)	<0.001 **	2.72	<0.001 **	2.43	0.42	0.70
[La^−^] (mmol·L^−1^)	2.5 ± 0.6 (2.0, 2.9)	4.7 ± 1.1 (3.9, 5.5)	4.4 ± 1.0 (3.7, 5.1)	<0.001 **	2.48	<0.001 **	2.27	0.63	0.27

Mean ± SD values (95% confidence limits). [La^−^] = blood lactate concentration. HR = heart rate. ** *p* ≤ 0.01.

**Table 2:** Mean ± SD values of the first 100 m time trial, biomechanical and psychophysiological variables assessed during experimental protocols: no-warm-up (NWU), typical warm-up (WU) and with post-activation potentiation (PAP) (n = 11).

				NWU vs. WU		NWU vs. PAP		WU vs. PAP	
	NWU	WU	PAP	*p*-Value	ES	*p*-Value	ES	*p*-Value	ES
T0–50 (s)	7.30 ± 0.68 (6.85, 7.64)	7.01 ± 0.58 (6.68, 7.34)	7.00 ± 0.62 (6.61, 7.39)	0.34	0.44	0.39	0.44	1.00	0.02
T50–100 (s)	8.69 ± 0.69 (8.20, 9.18)	7.66 ± 0.73 (7.13, 8.18)	7.66 ± 0.91 (7.01, 8.31)	0.03 *	1.39	0.04 *	1.23	1.00	0.00
T100 (s)	15.99 ± 0.96 (15.30, 16.68)	14.67 ± 1.29 (13.75, 15.60)	14.66 ± 1.52 (13.58, 15.74)	0.01 ***	1.12	0.02 *	1.03	1.00	0.01
T0–50 SF (Hz)	1.97 ± 0.19 (1.84, 2.11)	2.08 ± 0.14 (1.97, 2.18)	2.04 ± 0.13 (1.95, 2.13)	0.12	0.64	0.55	0.42	0.15	0.28
T50–100 SF (Hz)	1.72 ± 0.21 (1.57, 1.87)	1.89 ± 0.11 (1.81, 1.96)	1.91 ± 0.10 (1.84, 1.98)	0.05 *	1.02	0.03 **	1.17	0.77	0.18
T0–50 SL (m)	3.51 ± 0.32 (3.28, 3.75)	3.47 ± 0.34 (3.23, 3.71)	3.54 ± 0.36 (3.3, 3.86)	0.74	0.12	1.00	0.08	0.01 **	0.19
T50–100 SL (m)	3.40 ± 0.33 (3.16, 3.63)	3.51 ± 0.42 (3.21, 3.81)	3.47 ± 0.42 (3.17, 3.77)	0.10	0.28	0.42	0.18	0.48	0.09
HR (bpm)	148 ± 24 (131, 165)	156 ± 22 (140, 172)	162 ± 18 (149, 175)	1.00	0.33	0.43	0.64	0.84	0.29
[La^−^]_peak_ (mmol·L^−1^)	7.6 ± 1.8 (6.3, 8.8)	8.5 ± 1.3 (7.5, 9.4)	8.9 ± 1.5 (7.8, 10.1)	0.38	0.56	0.32	0.75	1.00	0.27
RPE	6 ± 2 (5, 7)	7 ± 1 (6, 8)	7 ± 1 (6, 7)	1.00	0.35	0.76	0.30	1.00	0.08

Mean ± SD values (95% confidence limits). HR = heart rate. [La^−^] = blood lactate concentration. RPE = ratings of perceived exertion. ** *p* ≤ 0.01 and * *p* ≤ 0.05.

**Table 3:** Mean ± SD values of the second 100 m time-trial, biomechanical and psychophysiological variables assessed during experimental protocols: no-warm-up (NWU), typical warm-up (WU) and with post-activation potentiation (PAP) (n = 11).

				NWU vs. WU		NWU vs. PAP		WU vs. PAP	
	NWU	WU	PAP	*p*-Value	ES	*p*-Value	ES	*p*-Value	ES
T0–50 (s)	7.16 ± 0.59 (6.73, 7.58)	7.03 ± 0.56 (6.63, 7.43)	6.97 ± 0.59 (6.55, 7.39)	0.80	0.22	0.32	0.31	1.00	0.10
T50–100 (s)	7.76 ± 0.61 (7.33, 8.20)	7.70 ± 0.82 (7.11, 8.29)	7.78 ± 0.98 (7.08, 8.48)	1.00	0.08	1.00	0.02	1.00	0.09
T100 (s)	14.92 ± 1.16 (14.09, 15.75)	14.73 ± 1.36 (13.76, 15.70)	14.75 ± 1.52 (13.67, 15.84)	1.00	0.14	1.00	0.12	1.00	0.01
T0–50 SF (Hz)	1.98 ± 0.16 (1.87, 2.10)	2.04 ± 0.09 (1.97, 2.11)	2.02 ± 0.12 (1.94, 2.10)	0.42	0.46	0.82	0.27	1.00	0.18
T50–100 SF (Hz)	1.88 ± 0.14 (1.77, 1.98)	1.89 ± 0.12 (1.80, 1.97)	1.86 ± 0.13 (1.77, 1.95)	1.00	0.07	1.00	0.14	1.00	0.23
T0–50 SL (m)	3.56 ± 0.32 (3.33, 3.79)	3.51 ± 0.32 (3.28, 3.74)	3.59 ± 0.38 (3.32, 3.86)	0.74	0.15	1.00	0.08	0.18	0.22
T50–100 SL (m)	3.47 ± 0.39 (3.19, 3.75)	3.49 ± 0.38 (3.22, 3.75)	3.52 ± 0.47 (3.18, 3.85)	1.00	0.05	1.00	0.11	1.00	0.07
HR (bpm)	164 ± 10 (157, 171)	161 ± 29 (140, 182)	172 ± 20 (158, 186)	1.00	0.15	0.25	0.51	0.63	0.43
[La^−^]_peak_ [mmol·L^−1^]	10.6 ± 1.6 (9.5, 11.7)	11.7 ± 1.6 (10.6, 12.8)	11.7 ± 1.9 (10.4, 13.0)	0.16	0.66	0.43	0.60	1.00	0.00
RPE	7 ± 2 (6, 8)	7 ± 1 (6, 8)	7 ± 1 (7, 8)	1.00	0.06	1.00	0.14	0.84	0.23

Mean ± SD values (95% confidence limits). HR = heart rate. [La^−^] = blood lactate concentration. RPE = ratings of perceived exertion.
